# Evaluation of the Relationship Between Facial Measurements and Esthetic Evaluation of the Face in Patients With a Vertical Growth Pattern

**DOI:** 10.7759/cureus.77021

**Published:** 2025-01-06

**Authors:** Marwa Ali Albitar, Ahmad S Burhan, Mohammad Y. Hajeer, Ossama Aljabban, Mowaffak A Ajaj, Fehmieh R Nawaya, Ahmad Salim Zakaria

**Affiliations:** 1 Department of Orthodontics, Faculty of Dentistry, University of Damascus, Damascus, SYR; 2 Department of Endodontics and Restorative Dentistry, Faculty of Dentistry, University of Damascus, Damascus, SYR; 3 Department of Pediatric Dentistry, Faculty of Dentistry, Syrian Private University, Damascus, SYR; 4 Department of Orthodontics, School of Dental Sciences, University Sains Malaysia, Kota Bharu, MYS

**Keywords:** attractiveness, facial esthetics, facial measurements, facial proportions, facial width, frontal photographs, lateral photographs, panel of raters, vertical growth pattern malocclusion

## Abstract

Background: Soft tissue specifications and facial values ​​vary depending on the underlying skeletal structures. To achieve the ideal treatment result and patient satisfaction, one must know the attractive soft tissue specifications compatible with each type of malocclusion. This study aims to analyze the facial measurements that contribute to perceived facial attractiveness in patients with vertical growth patterns and skeletal class I malocclusion, focusing on gender-specific differences.

Methodology: A panel of 30 laypersons, including raters from both genders equally, aged 19-24 years, evaluated extraoral photographs taken before the treatment of 60 patients (evenly divided between males and females employing a disproportionate stratified sampling method through a computer-generated list) with skeletal class I malocclusion, vertical growth pattern based on the Bjork sum, aged 18-25 years (with an average age of 22 ± 1.53 years), with the photographs taken in three positions (frontal relaxed, frontal during a smile, and relaxed profile). The raters utilized the visual analog scale (VAS) to assign an esthetic quality score to each photograph. Based on the average esthetic scores of each photo, two groups were created: the most attractive group, which received the highest esthetic score, and the least attractive group, which received the lowest esthetic score. After selecting 12 patients for each group, the angles and proportions of the frontal and lateral photos were calculated, and the results were compared between the two groups using an independent-sample t-test to see any significant differences.

Results: The most attractive females had a significantly lower value of mouth width to lower facial height than the least attractive females (P = 0.039). In addition, the most attractive males had a substantially greater value of facial convexity angle than the least attractive males (P = 0.041). Regarding other profile and frontal variables, no statistically significant differences existed between the most and least attractive males and females.

Conclusions: In patients with vertical growth pattern malocclusion, it is important to consider the chin protrusion of male patients during treatment planning and diagnosis because it enhances masculine features in these patients, as well as the lower facial height of female patients.

## Introduction

Orthodontic treatment aims to improve facial esthetics and the soft tissue profile by changing the underlying skeletal hard tissue and dentoalveolar structures [1]. Patients nowadays prioritize the esthetic outcome of orthodontic treatment over function and occlusion [2]. The idea of facial attractiveness is strongly linked to culture, media, and ethnic factors, as well as fashion [3,4]. Assessments of facial attractiveness are intricate and differ significantly among individuals and different cultures, as stated in Margaret Hungerford’s classic statement, "Beauty is in the eye of the beholder" [5]. When planning orthodontic treatment, it is important to consider the patient’s esthetic preferences and societal norms [6]. However, not enough information is available on the correlations between standard cephalometric values and perceived face beauty [7].

Limited soft tissue esthetic evaluations through cephalometric radiographic records led to a quest for numerical and proportional evaluations using photographic records [8,9]. Because of its low cost, availability of equipment, and time-saving advantages, photogrammetry is advised for large epidemiological research [10]. In addition, photographs are considered the most accurate representation of an individual's daily perception of facial features [11]. It is important to note that the angle at which a face is viewed can impact its perception attractiveness [12]. Therefore, orthodontists often rely on frontal, frontal smiling, and lateral photographs to diagnose issues, analyze facial esthetics, and plan treatments [13]. Stereophotogrammetry has been suggested to evaluate patients with dentofacial deformities. However, its use is restricted because of the high cost of the devices [14,15]. The interest in the analysis of soft tissue two-dimensionally is still under common use in orthodontic research. Mortada et al. compared the most and least attractive individuals in each gender for patients with skeletal class II division 1 malocclusion regarding facial angles and proportions that affect facial esthetics. After analyzing the frontal and profile characteristics, they concluded that the most attractive females were comparable to the least attractive. Meanwhile, the chins of the most attractive males protruded more than those of the least attractive [16].

Harrar et al., through their systematic review, attempted to answer the question: Is the art of assessment grounded in science, and if so, can it be measured? This study aimed to conduct an evidence assessment on the validity of quantitative facial measurements in rating beauty to support practitioners in their daily esthetic practices. They concluded that despite metrics like symmetry and the golden ratio that quantify the beauty of a component as a whole, we still have a long way to go before we can define quantitative beauty [17].

When patients with skeletal disorders are treated, orthodontists try to achieve the ideal esthetic values ​​of the extraoral soft tissues that have been established for a long time [6]. However, these standard values ​​depend on the presence of normal skeletal structures, which these patients do not have [18]. Therefore, dealing with these patients according to their skeletal structures is necessary. This research aims to investigate the measurements that make the faces of patients with a vertical growth pattern in skeletal class I malocclusion (in each gender) more receptive and attractive.

## Materials and methods

Study design and settings

This was a cross-sectional study for descriptive and analytical purposes. The collected data were based on patients’ photographs. This study was conducted at the Department of Orthodontics, Faculty of Dentistry, University of Damascus (Damascus, Syria) between March 2022 and June 2023. The Local Research Ethics Committee of the Faculty of Dentistry, University of Damascus, approved this work on February 15, 2022 (ID: UDDS-999-37072022/SRC-1450). Since this was not a clinical trial, the research protocol was not registered at clinical trial registries.

Sample size calculation

Using Minitab® version 21 (Minitab Inc., State College, Pennsylvania, USA), the sample size is calculated once the hypotheses are given, whereas the researcher did not use any of the commonly available statistical equations for estimating the sample size. One of the main ratios used in assessing facial length was "the lower facial height/middle and lower facial height." A difference of 4.5% between the two groups undergoing evaluation (i.e., the most and least attractive individuals) was considered clinically significant, according to an agreement between members of the research team. This variable's standard deviation from an earlier study was 1.02 degrees [16]. Employing a two-sample t-test with a power of 0.85 and an alpha level of 0.05, the required sample size was 12 persons in each group (i.e., 24 patients). However, to differentiate between the most attractive people who received the highest evaluation scores and the less attractive people who received the lowest, it was preferable to have a separation of about six to 10 people placed in the middle between the most attractive and the least attractive. Therefore, it was decided to add six patients to the required twenty-four to each group according to gender to reach a sample size of 30 patients.

Sample collection and patient recruitment

After an assessment of 136 patients attending the Department of Orthodontics at the Faculty of Dentistry at Damascus University, it was found that 92 patients met the inclusion criteria. Each patient received an information leaflet explaining the purpose and methods of the study. Eventually, 83 patients gave their permission to take part in the research and gave their informed consent. Afterward, a computer-generated sampling technique was employed to randomly choose 60 out of the 83 prospective patients, guaranteeing a balanced distribution of males and females, specifically 30 males and 30 females.

The patients' inclusion criteria included having skeletal class I, vertical growth pattern with Bjork sum between 402 and 406, and being between the ages of 18 and 25 age. The following were used as exclusion criteria: history of past teeth extractions (apart from third molars), previous orthodontic or prosthodontic therapy, and previous face esthetic surgery (such as lip augmentation or rhinoplasty), craniofacial syndrome, and severe skeletal vertical growth pattern (Bjork>406)

Photography method

With a Casio Exilim EX-H15 camera (Casio Exilim EX-H15; Casio Computer Co. Ltd., Japan) 14.1 megapixels, 24-240 mm macro lens, a total of 60 patients were photographed based on the natural daylight in three different positions: profile relaxed, frontal relaxed, and frontal during a smile [19]. Patients were asked to stand straight and place their arms freely on both sides of their bodies [20] to achieve the natural head position (NHP), as described by Moorrees et al. [21]. To attain this position, a mirror with dimensions of 24 × 55 cm with a movable base that can be moved to suit the patient's height was placed across from the patient at a distance of 120 cm [22].

Patients were instructed to rest their lips and focus on the horizontal line drawn on the mirror, symbolizing the line passing between the pupils of the eyes and parallel to the ground. White backgrounds were used when taking photographs to prevent the influence of the surrounding colors on the esthetic evaluation of the photographs. Utilizing a camera mount, the camera was positioned 150 cm from the Nasion point in frontal photos and the Porion point in profile photos. To eliminate any bias resulting from facial traits like skin texture, blemishes, eye color, eyebrow size, and position, and in some cases, makeup/jewelry, using the Photos program version 2018.18011.15918.0 (Photos, Microsoft Corp., Seattle, WA, USA), all patient photographs were turned into black and white to avoid how the surrounding colors affected how visually appealing the photos were.

Evaluation panel and raters' recruitment

A printed information sheet was distributed to collect a sample of evaluators, and the research objectives and evaluation method were explained to students of the Faculty of Engineering (Civil, Electrical Communications, and Information Technology) at the University of Damascus. After that, 74 pupils consented to take part in the assessment. Then, utilizing a disproportionate stratified sampling method, 30 students were selected through a computer-generated list to guarantee a balanced distribution of males and females (15 males and 15 females). Inclusion criteria for raters were undergraduate students, whose ages ranged between 19 and 24 years; they were not conversant with orthodontics or esthetics and had no relationship with the patients. Thirty laypeople evaluated the 60 patient photo sets.

Evaluation procedures of the patients’ photos

The photographs of the patients were shuffled and randomly inserted into a Microsoft PowerPoint 2016 slide show (Microsoft Corp., Seattle, WA, USA) with a fixed image size of 13.2 x 8.5 cm. Each slide, including a patient set, was displayed for 10 seconds on a laptop (cp2033dx-17, Hewlett and Packard, Palo Alto, California, USA), with an interval of 10 seconds after every 15th slide in a silent and comfortable room. The evaluation method was explained to the evaluators, and guidance was provided regarding the visual analog scale (VAS), which has a range of 0 to 100 mm on the rating sheet, to rate the appearance of the face [16]. A score of 50 indicated the average level of facial esthetics. Each patient photo set's mean evaluation score was determined, and the 60 patients were rated based on their means. For each gender, the 12 most and 12 least attractive photos were chosen.

Photographic analysis of facial soft tissues

**Figure 1:**
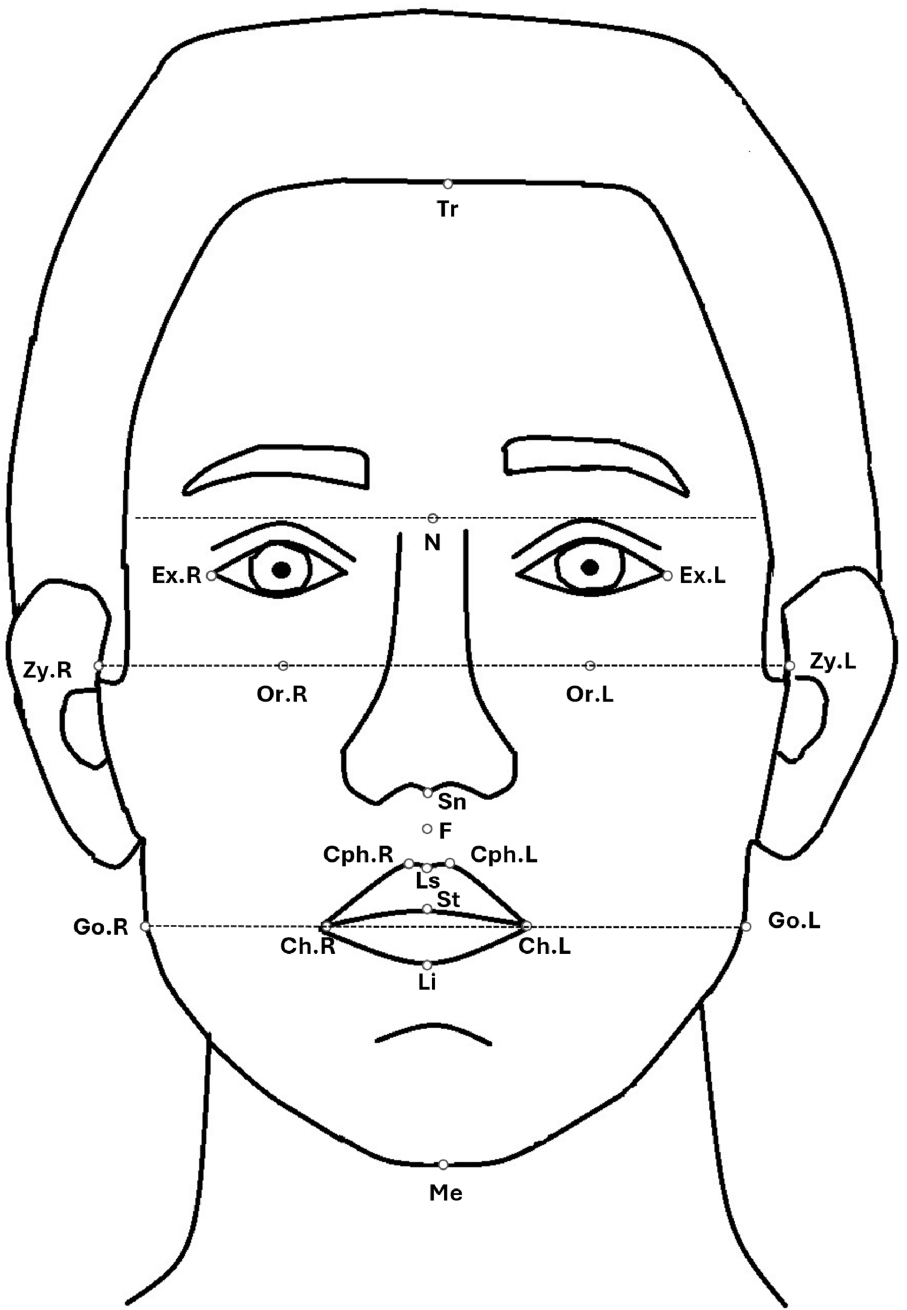
Landmarks positioned on the frontal photographs of the patient's face. The explanations of the landmark abbreviations are given in Table 1. Image credit: Marwa Ali Albitar

**Figure 2:**
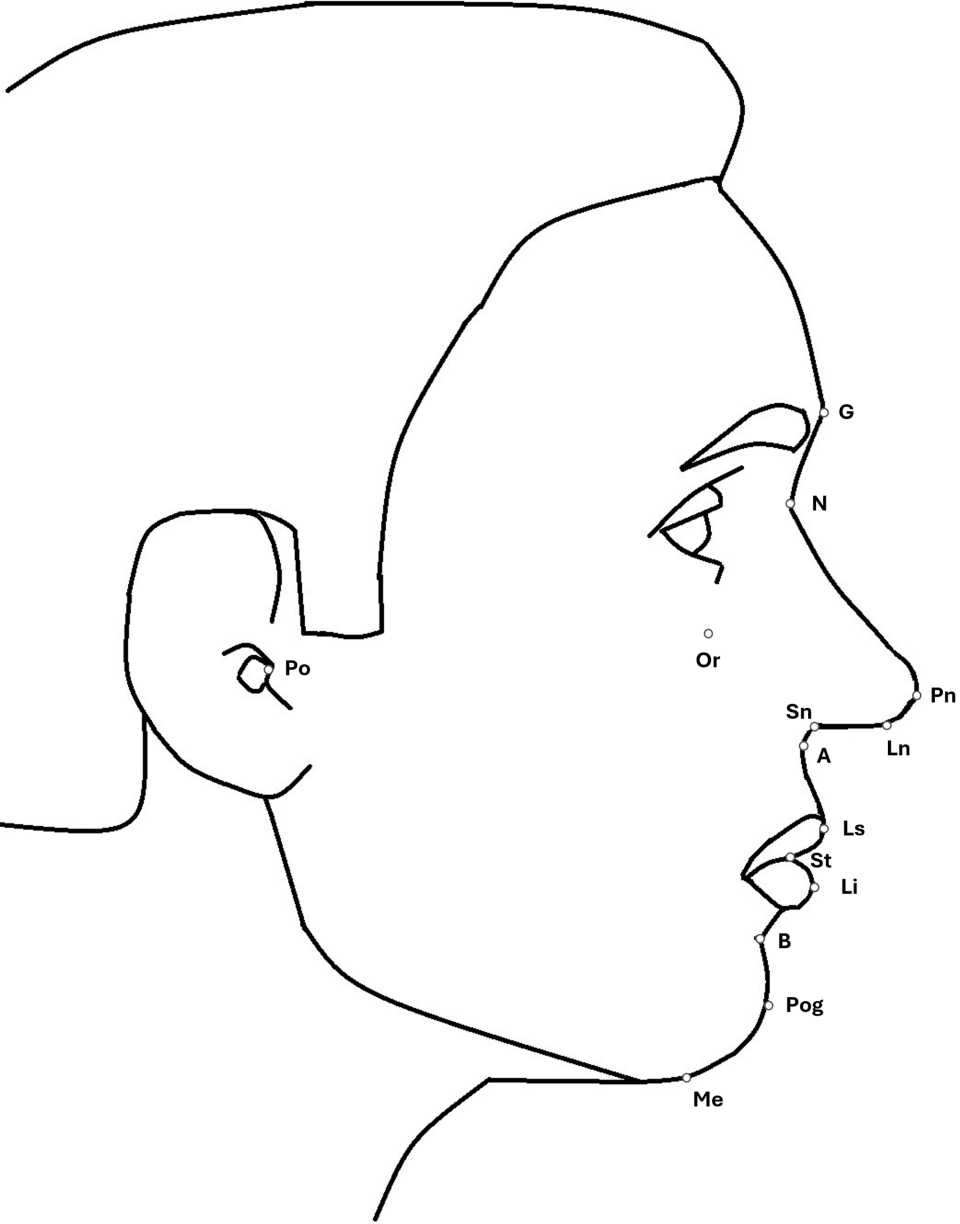
Landmarks positioned on the lateral photograph of the patient's face. The explanations of the landmark abbreviations are given in Table 2. Image credit: Marwa Ali Albitar

The evaluation included frontal and lateral photographs of the patients' faces taken at rest. The frontal photograph had 13 landmarks. The definitions of the landmarks used in the frontal photograph are given in Table 1 and shown in Figure 1 [23,24]. Twelve landmarks on the lateral photograph are also used. The definitions of these landmarks used in the frontal photographs are given in Table 2 and shown in Figure 2 [23].

**Table 1:** Definitions of the landmarks used on the frontal photograph. The landmark definitions are taken from * Kiekens et al. [23] and † Farkas et al. [24].

Landmark	Definitions
Tr (trichion)*	Point at the hairline
N (nasion)†	Point at the middle of the line that connects the superior palpebral sulci's highest points
Ex (exocanthion)*	Lateral eye contact
Or ( infraorbitale)†	The infraorbital point is situated under the lower eyelid, extending to the entrance of the eye when the eyes are at rest and facing directly ahead.
Sn (subnasale)*	Point at the platform of the columella and superior lip connection
F (philtrum) †	Point at the superior labial sulcus
Ls (labrale superior)*	Point in the center of the vermilion margin of the lip upper
Li (labrale inferior)*	Point in the center of the vermilion margin of the lip lower
St (stomion)*	Point of connection of the upper lip with the lower lip
Ch (cheilion)*	Points on the angles of the mouth
Cph (crista-philtrum) †	Points at the top of the vermilion margin of the superior lip
Me (menton)*	Lowest point in the middle of the soft tissues of the chin
Zy (zygion)*	Point of intersection between the line connecting the two infraorbital points and the outer border of the face on both sides
Go (Gonion)*	Constructed points at a stomion

**Table 2:** Definitions of the landmarks used on the lateral photograph The landmark definitions are taken from Kiekens et al. [23].

Landmark	Definitions
G (Glabella)	Point at the upper margin of the eyebrows
N (nasion)	Most hollow point between the nose and the forehead
Sn (subnasale)	Point at the platform of the columella and the superior lip connection
Ln (Lowest Nose Point)	Lowest point on the convexity of the nasal column
A	Most extreme point at the upper labial sulcus
B	Most extreme point at the lower labial sulcus
Ls (labrale superior)	Point in the center of the vermilion edge of the upper lip
St (stomion)	Point of connection of the upper lip with the lower lip
Li (labrale inferior)	Point in the center of the vermilion edge of the lip lower
Pog (Pogonion)	Most prominent point on the chin
Me (menton)	Lowest point in the middle of the soft tissues of the chin
Po (porion)	Point at the highest point on the tragus

Thirteen ratios and four angles on frontal photos were calculated, and the definition of these ratios and angles is given in Table 3 [25-27]. Eight angles on lateral photographs were measured manually, defined in Table 4 [9,28,29]. It was determined that each of these measurements was necessary to quantify the soft-tissue esthetics of the face. Some variables were attached to soft tissue harmony, such as the nasolabial angle (Ln.Sn.Ls) and the labiomental angle (Li.B.Pog), whereas some variables concerned with facial symmetry, such as the angle between the facial midline and subnasale-menton plane (N.F-Sn.Me). The computation that follows was utilized to ascertain the proportion of values that differ between the groups that are most and least attractive for every variable: (λ1- λ2)/ λ2 × 100, where λ1 denotes the variable’s mean value inside the most attractive group and λ2 denotes the variable’s mean value inside the least attractive group.

**Table 3:** Definitions of the variables used in the analysis of frontal photographs Variable definitions are taken from * Farkas et al. [25], † Koury and Epker et al. [26], and ‡ Morosini et al. [27].

Variable	Definition
N.Sn/N.Me *	Nasal length/middle and lower facial length
Ls.St/St.Li *	Superior vermilion length /inferior vermilion length
Ls.St/Sn.St *	Superior vermilion length / superior lip length
Sn.Me/N.Me *	Lower facial length /middle and lower facial length
Sn.Me/Tr.Me *	Lower facial length /overall facial length
Sn.St/ChR.ChL †	Superior lip length /mouth breadth
ChR.ChL/Sn.Me †	Mouth breadth /lower facial length
Cph.Li/ChR.ChL †	Superior and inferior vermilion length /mouth breadth
Sn.St/SnMe †	Superior lip length /lower facial length
ZyR.ZyL/Tr.Mn †	Facial breadth / overall facial length
N.Me/ZyR.ZyL †	Middle and lower facial length /facial breadth
Sn.Me/ZyR.ZyL †	Lower facial length /facial breadth
ChR.ChL/ZyR.ZyL †	Mouth breadth /facial breadth
ZyR.Me.ZyL ‡	Upper facial breadth angle
GoR.Me.GoL ‡	Mandibular breadth angle
ExR.Me.ExL ‡	Facial slot modified angle
N.F-Sn.Me ‡	Facial symmetry angle( angle between the facial midline and subnasal menton line)

**Table 4:** Definitions of the variables used in the analysis of lateral photographs. Variable definitions are taken from * Nguyen and Turley [28], † Holdaway [29], and ‡ Peck and Peck [9].

Variable	Definition
Ln.Sn.Ls *	Nasolabial angle
Li.B.Pog *	Labiomental angle
Sn.Ls-B.Li *	Angle between the upper and lower lips
G.Sn.Pog *	Facial convexity angle
Po.N.Pog †	Mandibular prominence (angle between the soft-tissue Pogonion-Nasion plane and the soft tissue facial plane).
ANB †	Soft-tissue ANB
Sn.Po.Me ‡	Inferior facial third angle
N.Po.Pog ‡	Total vertical angle

Assessment of systematic and random error

After a month, the same researcher examined the variables again on the frontal and lateral photos of 20 randomly chosen patients. A paired sample t-test was used to compare the first and second readings to look for systematic errors. The intraclass correlation coefficients (ICCs) were used to assess the intraobserver reliability (i.e., random error).

Evaluation of inter-rater reliability

Six evaluators were randomly selected, and they were asked to re-evaluate a group of patients’ photos (10 patients) one month after the end of the overall evaluation process; the paired-sample t-test for correlated samples was conducted to examine the significance of the differences in the average ratings to compare the first and second evaluation scores.

Statistical analysis

The statistical analysis was performed using IBM SPSS Statistics for Windows, Version 27.0 (released 2020, IBM Corp., Armonk, NY). The Shapiro-Wilk test was used to examine the data distribution's normality. The differences between the most and least attractive groups were assessed using an independent-sample t-test. A significance threshold of 0.05 was chosen.

## Results

Error of the method and reliability of the measurements

For both frontal and lateral measurements, there were no statistically significant differences in the measurements made during the first and second assessment times (P > 0.05). The great reliability of the measuring procedure was demonstrated by the interclass correlation coefficients, which fell between 0.932 and 0.998. The findings of the paired sample t-tests point to the small and inconsequential systematic errors, as shown in Table 5 and Table 6.

**Table 5:** The error of method and the intra-observer reliability for frontal variables. *Mean differences between the two sets of measurements, †: P-value for paired-sample t-test, ‡: P-value for ICC. ICC: intra-class correlation; LB: lower bound; UB: upper bound; N-Sn/N-Me: nasal length/middle and lower facial length; Ls-St/St-Li: superior vermilion length/inferior vermilion length; Ls-St/Sn-St: superior vermilion length/superior lip length; SnSt/ChR-ChL: superior lip length/mouth breadth; Cph-Li/ChR-ChL: superior and inferior vermilion length/mouth breadth; Sn-St/Sn-Me: superior lip length/lower facial length; Sn-Me/N-Me: lower facial length/middle and lower facial length; Sn-Me/Tr-Me: lower facial length/overall facial length; ChR-ChL/Sn-Me: mouth breadth/lower facial length; ZyR.ZyL/Tr-Me: facial breadth/overall facial length; N-Me/ ZyR.ZyL: middle and lower facial length/facial breadth; Sn-Me/ZyR.ZyL: lower facial length/facial breadth; ChR-ChL/ ZyR.ZyL: mouth breadth/facial breadth; ZyR.Me.ZyL: upper facial breadth angle; GoR.Me.GoL: mandibular breadth angle; ExR.Me.ExL: facial slot modified angle; N.F-Sn.Me: facial symmetry angle (angle between the facial midline and the subnasal-menton line).

Variables	First measurements	Second measurements	Mean difference*	P-value†	ICC	95% CI		P-value‡
						LB	UB	
N.Sn/N.Me	48.59	49.48	2.38	0.754	0.964	0.904	0.987	<0.001
Ls.St/St-Li	57.87	58.01	0.57	0.874	0.998	0.996	0.999	<0.001
Ls.st/Sn.St	27.61	28.44	0.83	0.907	0.932	0.823	0.975	<0.001
Sn.St/ChR.ChL	40.53	40.94	0.41	0.762	0.998	0.993	0.999	<0.001
Cph.Li/ChR.ChL	31.66	32.61	0.95	0.697	0.982	0.950	0.993	<0.001
Sn.St/SnMe	34.64	33.22	1.01	0.831	0.967	0.908	0.988	<0.001
Sn.Me/N.Me	52.01	52.53	0.52	0.719	0.980	0.945	0.993	<0.001
Sn.Me/Tr.Me	38.91	37.28	0.37	0.745	0.941	0.838	0.979	<0.001
ChR.ChL/Sn.Me	85.66	86.37	1.71	0.614	0.964	0.900	0.987	<0.001
ZyR.ZyL /Tr.Me	68.42	69.02	2.38	0.803	0.945	0.847	0.980	<0.001
N.Me/ZyR.ZyL	92.97	93.70	2.73	0.771	0.950	0.982	0.993	<0.001
Sn.Me/ZyR.ZyL	48.00	48.88	1.88	0.954	0.994	0.984	0.998	<0.001
ChR.ChL/ ZyR.ZyL	39.72	40.12	0.40	0.692	0.980	0.944	0.993	<0.001
ZyR.Me.ZyL	73	73.73	0.73	0.649	0.989	0.971	0.996	<0.001
GoR.Me.GoL	103.28	101.44	5.164	0.746	0.973	0.925	0.990	<0.001
ExR.Me.ExL	41.14	41.96	0.822	0.832	0.996	0.988	0.998	<0.001
N-F.Sn-Me	1.712	1.748	0.034	0.916	0.995	0.986	0.998	<0.001

**Table 6:** Error of method and intra-observer reliability for lateral variables. *Mean differences between the two sets of measurements, †: P-value for paired-sample t-test, ‡: P-value for ICC. ICC: intra-class correlation; LB: lower bound; UB: upper bound; Ln.Sn.Ls: nasolabial angle; Li.B.Pog: labiomental angle; Sn-Ls.B-Li: angle between the upper and lower lips; Po.N.Pog: mandibular prominence (angle between the soft-tissue Pogonion-Nasion plane and the soft-tissue facial plane); ANB: soft-tissue ANB; G.Sn.Pog: facial convexity angle; Sn.Po.me: inferior facial third angle; N.Po.Pog: total vertical angle.

Variables	First measurements	Second measurements	Mean difference*	P-value†	ICC	95% confidence interval		P-value‡
						LB	UB	
Ln.Sn.Ls	110.78	111.959	2.215	0.729	0.998	0.993	0.999	<0.001
Li.B.Pog	128.14	128.765	2.542	0.987	0.996	0.988	0.999	<0.001
Sn-Ls.B-Li	139.07	138.198	0.47	0.890	0.993	0.976	0.998	<0.001
Po.N.Pog	71.964	72.040	2.158	0.730	0.997	0.989	0.999	<0.001
ANB	7.678	8.543	0.153	0.863	0.992	0.972	0.998	<0.001
G.Sn.Pog	0.88	0.891	0.008	0.723	0.997	0.990	0.999	<0.001
Sn.Po.Me	40.428	39.361	0.404	0.839	0.993	0.976	0.998	<0.001
N.Po.Pog	52.75	53.027	0.527	0.843	0.998	0.993	0.999	<0.001

Evaluation of inter-rater reliability

The evaluating inter-rater reliability using the paired-sample t-test showed that the P-value for all evaluations was greater than 0.05, meaning there are no statistically significant differences between the average of the first and second evaluation scores. Therefore, only one evaluation was deemed sufficient to determine the degree of facial attractiveness of patients in the current research.

Baseline sample characteristics

The most and least attractive groups comprised 24 males and 24 females (12 in each group). The mean age of the most and least attractive males was 23.7 ± 0.96 and 22.61 ± 0.07 years, respectively. The mean age groups of the most and least attractive females were 19.21 ± 1.52 and 21.83 ± 1.43 years, respectively. No dropout occurred; therefore, the statistical analysis included all the patients.

Main findings of the variable measurements

Regarding the frontal variables between the most and least attractive groups, the most attractive females had a significantly lower value of mouth width/lower facial height (ChR.ChL/Sn.Me) than the least attractive females (P = 0.039). The mean values of this ratio were 75.81% in the most attractive and 82.19% in the least attractive females. On the other hand, there was no significant difference between the most and least attractive males regarding any of the frontal variables (Table 7).

**Table 7:** Descriptive statistics of the measurements of the frontal variables with the p-values of significance testing between the two groups Employing independent samples t-test, *p-value significant at P < 0.05. SD: standard deviation; MA: most attractive; LA: least attractive; N-Sn/N-Me: nasal length /middle and lower facial length; Ls-St/St-Li: superior vermilion length/inferior vermilion length; Ls-St/Sn-St: superior vermilion length/superior lip length; SnSt/ChR-ChL: superior lip length/mouth breadth; Cph-Li/ChR-ChL: superior and inferior vermilion length/mouth breadth; Sn-St/Sn-Me: superior lip length/lower facial length; Sn-Me/N-Me: lower facial length/middle and lower facial length; Sn-Me/Tr-Me: lower facial length/overall facial length; ChR-ChL/Sn-Me: mouth breadth/lower facial length; ZyR.ZyL /Tr-Me: facial breadth/overall facial length; N-Me/ ZyR.ZyL: middle and lower facial length /facial breadth; Sn-Me/ ZyR.ZyL: lower facial length/facial breadth; ChR-ChL/ ZyR.ZyL: mouth breadth/facial breadth; ZyR.Me.ZyL: upper facial breadth angle; GoR.Me.GoL: mandibular breadth angle; ExR.Me.ExL: facial slot modified angle; N.F-Sn.Me: facial symmetry angle (angle between the facial midline and the subnasal-menton line).

Variables	MA females		LA females		P-value†	MA males		LA males		P-value†
	Mean	SD	Mean	SD		Mean	SD	Mean	SD	
N.Sn/N.Me	47.4	2.43	47.25	4.05	0.949	44.21	2.66	42.54	4.82	0.578
Ls.St/St.Li	42.16	17.88	50.75	19.08	0.387	52.73	17.23	47.33	14.91	0.711
Ls.st/Sn.St	20.56	6.9	21.34	8.17	0.663	25.98	3.5	23.85	3.43	0.322
Sn.St/ChR.ChL	48.93	8.23	43.76	5.51	0.154	48.22	6.87	50.33	6.06	0.739
Cph.Li/ChR.ChL	33.74	6.71	28.91	6.76	0.189	37.65	8.39	36.92	6.43	0.874
Sn.St/SnMe	36.29	5.12	35.76	4.19	0.748	31.28	1.22	36.46	3.8	0.061
Sn.Me/N.Me	52.37	2.87	50.72	4.44	0.21	54.3	2.98	55.01	3.19	0.775
Sn.Me/Tr.Me	37.98	7.56	37.89	7.23	0.854	36.38	1.98	37.37	2.18	0.64
ChR.ChL/Sn.Me	75.81	6.19	82.19	4.221	0.039*	65.18	6.54	74.51	17.72	0.394
ZyR.ZyL/Tr.Me	68.43	4.76	67.97	5.76	0.518	67.43	5.56	66.94	5.29	0.088
N.Me/ZyR.ZyL	91.45	8.43	95.16	3.65	0.231	99.54	14.23	89.33	18.35	0.413
Sn.Me/ZyR.Zyl	48.86	4.89	47.51	3.6	0.833	55.61	5.99	49.53	11.43	0.396
ChR.ChL/ZyR.ZyL	31.89	6.32	37.45	4.33	0.518	33.98	7.43	37.07	6.29	0.463
ZyR.Me.ZyL	69.5	4.47	69.3	2.92	0.474	66.5	7.85	76.5	10.1	0.17
GoR.Me.GoL	99.5	3.54	101	2.85	0.948	96.2	8.3	107	10.6	0.162
ExR.Me.ExL	46.2	3.37	46.7	2.86	0.162	43.7	4.78	46	6.16	0.585
N-F.Sn-Me	1.5	1.06	1.37	1.06	0.754	0.75	0.95	1	0.81	0.705

Among the profile variables, the most attractive males had a significantly higher value of facial convexity angle (G.Sn.Pog) than the least attractive males (P = 0.041). The mean values of this angle for the most attractive and least attractive males were 166° and 158°, respectively (Table 8).

**Table 8:** Descriptive statistics of the measurements of the profile variables with the p-values of significance testing between the two groups. †Employing independent samples t-test, *p-value significant at p < 0.05. SD: standard deviation; MA: most attractive; LA: least attractive;  Ln.Sn.Ls: nasolabial angle; Li.B.Pog: labiomental angle; Sn-Ls.B-Li: angle between the upper and lower lips; Po.N.Pog: mandibular prominence (angle between the soft-tissue Pogonion-Nasion plane and the soft-tissue facial plane); ANB: soft-tissue ANB; G.Sn.Pog: facial convexity angle; Sn.Po.me: inferior facial third angle; N.Po.Pog: total vertical angle.

Variables	MA females		LA females		P-value†	MA males		LA males		P-value†
	Mean	SD	Mean	SD		Mean	SD	Mean	SD	
Ln.Sn.Ls	102	4.59	102	10.2	0.975	102	9.91	95	9.12	0.294
Li.B.Pog	135	10	129	9.3	0.256	139	7.67	133	13.7	0.459
Sn.Ls-B.Li	122	12.1	125	8.15	0.572	143	12.8	120	26.3	0.18
Po.N.Pog	72.5	5.31	71.1	6.85	0.661	71.5	3.1	70.2	0.49	0.458
ANB	7.12	2.35	8.25	1.75	0.297	6.25	1.5	8	0.81	0.086
G.Sn.Pog	161	8.46	158	10.2	0.453	166	4.42	158	5.03	0.041*
N.Po.Pog	61.3	5.47	61	5.07	0.889	62.5	6.35	63	2.44	0.888
Sn.Po.Me	33.7	4.02	32.3	3.42	0.474	33.2	3.86	35.5	1.29	0.311

However, regarding any of the profile factors, no statistically significant difference was found between the most and least attractive females.

## Discussion

According to our knowledge, this is the first cross-sectional study that examines the esthetic values ​​of facial soft tissues in patients with skeletal class I vertical growth patterns, and in each gender separately, at the frontal and lateral views. In addition, this study contributed to our understanding of cultural differences.

Methodology employed in this project

To guarantee that no aspect of growing would subsequently change the face esthetics, all of the subjects in this study were older than eighteen (patients' age was between 18 and 25 years). Experts must examine the soft tissues and understand the factors influencing the face's esthetic appeal to achieve facial equilibrium [30]. Bjork's sum was relied upon to determine the growth pattern because Bjork's analysis has been shown to be very useful when assessing facial characteristics through only a few measurements [31]. The photographs were used to determine the facial measures due to their dependability, capacity to be retaken when necessary, and long shelf life [32]. Shooting the photos in a consistent and repeatable location is necessary for accurate measurements. The images were thus taken in the natural head position [21].

Three distinct photo modes were used for the evaluation, making it possible for laypeople to evaluate the esthetics of the face thoroughly because displaying the images as a whole would create a more comprehensive picture of the patient [19]. Images were converted to black and white to avoid discrimination based on facial characteristics such as skin tone, spots, eyebrow color, and occasionally makeup or jewelry [33]. The VAS was employed to rate the photographs. According to Gould et al., the VAS was more relevant because it allowed the rater to assign a point within a continuous interval instead of having them choose from a predetermined list of categories [34].

The rater panel consisted of laypeople with no connection to the patients and no knowledge of esthetics or orthodontics. The rationale behind selecting this panel was the belief that the opinions of laypeople are the most impartial and objective [35]. Moreover, Kiekens et al. demonstrated that seven laypeople were sufficient to offer a reliable esthetic evaluation of photographs [23]. Each photo was evaluated thirty times to increase the study's conclusion accuracy. Furthermore, because gender and ethnic differences exist, it is not advisable to use absolute values in facial analysis [26]. As a result, facial measures were assessed using angles and ratios that could be redefined in a still appropriate way [13,23,25].

Frontal variables

Regarding the frontal variables, there was a significant decrease in the ratio of the mouth width to the lower facial height in the group of most attractive females by an amount (8.54%). Although this fundamental difference exists from a statistical standpoint, its clinical significance may be a point of difference where some practitioners may consider this percentage essential from a clinical standpoint while others do not. The current results corroborate the research conducted by Malkoc and Fidanciogl. Where the general public's perceptions indicate that there is a negative association between this percentage and the values of facial beauty [36]. Conversely, the findings of this trial disagreed with those reported by Kiekens et al.'s study, which did not discover a connection between this ratio and the attractiveness of the face. The variations can be attributed to cultural variables and the age range of the resident committees, which was 28 to 76 years old. Patients comprised teenagers aged 10 to 16 [23]. A study by Jang et al. found that the Miss Korea group had a greater value for this ratio and a smaller linear measurement of the lower facial length. The disagreement might result from different cultural preferences for esthetics and the control sample's selection process, which were based only on orthodontics criteria (normal face, straight profile, and palmar interlabial distance of less than 1 mm) rather than esthetic evaluation [37]. 

Conversely, no statistically significant differences regarding frontal factors between the most and least attractive males were found. These results are consistent with those of Kiekens et al., who found no connection between facial attractiveness and any of the prior characteristics [23]. In addition, in line with our findings, Wroblewska et al. evaluated research on variables that may affect face symmetry and concluded that when asymmetry falls within the population average, it does not significantly affect facial beauty [38].

However, contrary to this study's results, Penna et al. observed that the beautiful groups of each gender had a considerably larger ratio of upper lip height to upper vermilion height. They concluded that both genders' facial esthetics are significantly influenced by a fuller top lip [39]. One possible reason for the discrepancies between their and current findings is that their sample only included photos of the perioral region, not the entire facial view.

In addition, Malkoc and Fidanciogl discovered a negative correlation between facial esthetics and the ratios of mouth width to lower facial height, lower facial height to facial width, and facial height to facial width, which could be explained by the fact that comparing the study's variables to their ideal values. This finding contradicts our findings [36].

Profile variables

Regarding the frontal variables, there was a significant increase in the angle of facial convexity in the group of most attractive males by an amount (13.51%). This result suggests that chin protrusion enhances masculine features in men and helps mask undesirable characteristics by increasing facial length in patients with vertical growth patterns. That was in agreement with the Fortes et al. study, which showed that men with higher facial convexity angles were more attractive than those with lower facial convexities [40]. They also agreed with the results of the Thakral et al. study, which found that the males who received the highest scores in the attractiveness assessment had lower facial convexity [7]. In addition, these results corroborate those of Suphatheerawatr and Chamnannidiadha. Who found that among the changed profiles, men's straight and slightly convex profiles were deemed to be the most beautiful [41].

Malkoc and Fidanciogl, in contrast to the current results of this study, demonstrated no relationship between the adolescent's profile esthetics and the position of their lower jaw. The discrepancy might result from different study methodologies; Malkoc and Fidanciogl examined the two genders together, whereas our study examined the two genders independently [36]. The findings also contradict those of Sforza et al.'s study, which found no discernible differences between the most attractive men and a control sample group drawn from the general public. This could be attributed to cultural differences in esthetic preferences between the sample of this study and the current study, as well as the age of the sample study (15-17 years) [42].

On the other hand, there were no significant differences among the profile variables between the most and least attractive females, which may be explained by the evaluation of female profiles conducted amorphously without considering the specifics of the profiles. The results of this study differed from those of Jang et al. and Kim et al. Jang found that the Miss Korea group had a larger nasolabial angle, while Kim found that the Miss Korea group had a smaller Labiomental angle than the female group from the control sample from the general population. The cultural and ethnic disparities between the sample in the current study, which evaluated the Syrian race, and these two studies that assessed the Korean race may cause the discrepancy [37,43]. Furthermore, the current study's results were not the same as those of Malkoc and Fidanciogl, who found that attractive people had shallower labiomental sulcus depths. The discrepancy between their findings and the current findings is that in Malkoc and Fidanciogl's study, these angles were compared with their ideal values and were not examined independently for each gender [36].

According to the study, greater values of facial convexity angles in males and lower mouth-width-to-lower facial height ratios in females are linked to higher attractiveness among patients with vertical growth patterns. These results highlight the importance of including these factors in orthodontic diagnosis and treatment planning to improve facial esthetics. According to these findings, it is recommended to emphasize the necessity of functional treatment to modify growth patterns, advance the chin in young patients, and consider the possibility of chin genioplasty treatments in adult males.

Methodological strengths

This research adopted standardized photography and standardized lighting conditions to make photographic analysis more valid and reliable [21,44]. The evaluation was performed on photographs in three different modes (profile relaxed, frontal relaxed, and frontal during a smile), allowing laypersons to conduct a comprehensive esthetic assessment of the face as displaying these photos together would achieve a more general and thorough image of the patient [19]. Images were converted to black and white to eliminate factors affecting facial esthetics assessment, such as skin, eye, and hair color [33]. The VAS was employed to rate the photographs because this method is considered simple, applicable, easy to understand, and gives independent assessments of the objects [34]. The rater panel consisted of laypeople with no connection to the patients and no knowledge of esthetics or orthodontics, this panel was selected because laypersons’ opinions are considered realistic and unbiased [35].

Limitations of the current work

This study included class I malocclusion patients with vertical growth patterns and did not have different types of sagittal skeletal or horizontal growth patterns. This study relies only on laypeople's judgment. The current sample was selected from a single teaching hospital based exclusively on one race, and rather a small sample size and limited variable scope, restricting the findings' generalizability. Future research is suggested by including diverse ethnic/cultural groups and mixed evaluation panels (laypeople, orthodontists, and artists), increasing the sample size of patients, and including other major esthetic variables. Using 3D imaging or AI is expected to enhance esthetic analysis. Researching facial esthetics in patients with vertical and sagittal deviated growth patterns is suggested.

## Conclusions

The study highlights that among individuals with vertical growth patterns, lower mouth-width-to-lower facial height ratios in females, and increased facial convexity angles in males are associated with higher attractiveness. These findings emphasize the importance of incorporating these variables into orthodontic diagnosis and treatment planning to enhance facial esthetics. While no significant differences were observed for other variables in males or females, further research is warranted to investigate additional factors influencing facial attractiveness, particularly in diverse cultural and ethnic populations. Future studies should also explore other malocclusion types and utilize advanced 3D imaging for comprehensive esthetic evaluation.
